# Facing the COVID-19 pandemic – an assessment of students’ mental health and major coping strategies during the COVID-19 pandemic – an international study

**DOI:** 10.1192/j.eurpsy.2023.376

**Published:** 2023-07-19

**Authors:** A. A. Forma, K. H. Karakuła, R. Sitarz, D. Juchnowicz, J. Baj, J. Bogucki, J. Rog, M. L. Tee, C. A. Tee, J. T. Ly-Uson, M. S. Islam, M. T. Sikder, A. H. El-Monshed, A. Loutfy, M. F. H. Qureshi, M. Abbas, S. Taseen, M. Lakhani, C. Wang, X. Wan, Y. Tan, R. Pan, R. Ho, S. Jayakumar, S. Ilango, S. Kumar K, Á. A. Ruiz-Chow, A. Iturbide, D. D. González-Mille, L. P. Doan, H. Karakuła-Juchnowicz

**Affiliations:** ^1^Chair and I Department of Psychiatry, Psychotherapy, and Early Intervention; ^2^Department of Psychiatric Nursing; ^3^Chair and Department of Anatomy; ^4^Chair and Department of Organic Chemistry, Faculty of Pharmacy; ^5^Chair and I Department of Psychiatry, Psychotherapy, and Early Intervention, Medical University of Lublin, Lublin, Poland; ^6^College of Medicine, University of Philippines Manila, Manila, Philippines; ^7^Department of Public Health and Informatics, Jahangirnagar University, Dhaka, Bangladesh; ^8^Department of Psychiatric and Mental Health Nursing, Faculty of Nursing, Mansoura University, Mansoura; ^9^Department of Pediatric Nursing, Faculty of Nursing, Beni-Suef University, Beni-Suef, Egypt; ^10^Ziauddin Medical University, Ziauddin Medical University, Ziauddin; ^11^Karachi Medical and Dental College, Karachi, Pakistan; ^12^Faculty of Education, Huaibei Normal University, Huaibei; ^13^Anqing Normal University, Anqing Normal University, Anqing, China; ^14^Department of Psychological Medicine, National University of Singapore, Singapore; ^15^Department of Basic Medical Sciences, Al Majmaah University, Majmaah, Saudi Arabia; ^16^Madga Medical College and Research Insitute, Chennai, India; ^17^Centro Médico ABC, Centro Médico ABC, Mexico City, Mexico; ^18^Duy Tan University, Da Nang, Viet Nam

## Abstract

**Introduction:**

TDuring COVID-19 pandemic, it was noticed that it was students who were mostly affected by the changes that aroused because of the pandemic. The interesting part is whether students’ well-being could be associated with their fields of study as well as coping strategies.

**Objectives:**

In this study, we aimed to assess 1) the mental health of students from nine countries with a particular focus on depression, anxiety, and stress levels and their fields of study, 2) the major coping strategies of students after one year of the COVID-19 pandemic.

**Methods:**

We conducted an anonymous online cross-sectional survey on 12^th^ April – 1^st^ June 2021 that was distributed among the students from Poland, Mexico, Egypt, India, Pakistan, China, Vietnam, Philippines, and Bangladesh. To measure the emotional distress, we used the Depression, Anxiety, and Stress Scale-21 (DASS-21), and to identify the major coping strategies of students - the Brief-COPE.

**Results:**

We gathered 7219 responses from students studying five major studies: medical studies (N=2821), social sciences (N=1471), technical sciences (N=891), artistic/humanistic studies (N=1094), sciences (N=942). The greatest intensity of depression (M=18.29±13.83; moderate intensity), anxiety (M=13.13±11.37; moderate intensity ), and stress (M=17.86±12.94; mild intensity) was observed among sciences students. Medical students presented the lowest intensity of all three components - depression (M=13.31±12.45; mild intensity), anxiety (M=10.37±10.57; moderate intensity), and stress (M=13.65±11.94; mild intensity). Students of all fields primarily used acceptance and self-distraction as their coping mechanisms, while the least commonly used were self-blame, denial, and substance use. The group of coping mechanisms the most frequently used was ‘emotional focus’. Medical students statistically less often used avoidant coping strategies compared to other fields of study. Substance use was only one coping mechanism that did not statistically differ between students of different fields of study. Behavioral disengagement presented the highest correlation with depression (r=0.54), anxiety (r=0.48), and stress (r=0.47) while religion presented the lowest positive correlation with depression (r=0.07), anxiety (r=0.14), and stress (r=0.11).

**Conclusions:**

1) The greatest intensity of depression, anxiety, and stress was observed among sciences students, while the lowest intensity of those components was found among students studying medicine.

2) Not using avoidant coping strategies might be associated with lower intensity of all DASS components among students.

3) Behavioral disengagement might be strongly associated with greater intensity of depression, anxiety, and stress among students.

4) There was no coping mechanism that provided the alleviation of emotional distress in all the fields of studies of students.

**Disclosure of Interest:**

None Declared

